# The effect of ketogenic diets on neurogenesis and apoptosis in the dentate gyrus of the male rat hippocampus

**DOI:** 10.1186/s12576-020-00786-7

**Published:** 2021-01-19

**Authors:** Irfannuddin Irfannuddin, Siti Fazzaura Putri Sarahdeaz, Krisna Murti, Budi Santoso, Noriyuki Koibuchi

**Affiliations:** 1 Department of Physiology, Faculty of Medicine, Universitas Sriwijaya, Gedung FK Unsri, Jalan Dr. M. Ali Komplek RSMH, Palembang, 30126 Indonesia; 2 Department of Integrative Physiology, Gunma University Graduate School of Medicine, Maebashi, Gunma 371-8511 Japan

**Keywords:** Ketogenic diet, Time-restricted diet, High-fat diet, Ki-67, Caspase-3, Immunohistochemistry

## Abstract

Ketogenic diets (KD) have become popular diet to lose weight. However, the effect of such diets on brain function has not yet been clarified. Thus, we aimed to study the effects of KD on the neurogenesis and apoptosis in the dentate gyrus by assessing the expression of Ki-67 and Caspase-3. Rats (*n* = 24) were divided into four groups: control (normal diet), ketogenic diet (KD), time-restricted diet (TRD), and the combination of high-fat and time-restricted diet (CD) groups. The expression of Ki-67 in the TRD and CD groups was higher compared to others (*P* < 0.05), whereas no such difference was observed in the KD group. The number of Capase-3-positive cells decreased significantly in the TRD group (*P* < 0.05), but such decrease was not observed in the CD group. These results indicate that, although KD could be effective in reducing the body weight, possible adverse effect in the brain cannot be ignored.

## Background

Obesity is the accumulation of abnormal or excessive fat that may cause adverse outcomes. Management of obesity consists of interventions through diet and exercise [1]. One of the effective dietary interventions to treat obesity may be energy-restricted diet which contains fat as a main energy source. The most popular such diet is ketogenic diet (KD). KD is a diet that uses high fat as an energy source and reduces carbohydrate and protein constitutions. KD has been shown to reduce body mass index (BMI) and weight in obese patients [2]. Thus, KD has been considered one of the most effective methods to treat obese patients, although the precise mechanism has not yet been fully clarified. On the other hand, KD causes ketosis and reduced glucose supply to the brain, which mainly uses glucose as an energy supply. Thus, KD may alter the metabolism in the brain.

In spite of the fact that KD may be beneficial in losing weight, consumption of fat has been considered as a potential cause of cardiovascular diseases, although saturated fat has no significant effect to increase the cardiovascular disease [3]. In the brain, on the other hand, the cognitive function could be influenced by high-fat or sugar diets [4]. Furthermore, impaired adult neurogenesis may cause various abnormalities, such as declined cognitive functions and obesity [5]. Thus, we hypothesized that KD may also alter the cognitive function through altering the neurogenesis. Among brain areas showing neurogenesis throughout the life, we focused on the dentate gyrus of the hippocampus, which plays a critical role in controlling the cognitive functions [6]. In fact, a previous study showed that KD disrupts the neurogenesis [7]. In addition to altered neurogenesis, consumption of high fat for the long term has been thought to cause mitochondrial oxidative stress in the brain [8]. Increased mitochondrial stress may activate the pathway to induce apoptosis [9]. A previous study has shown that KD disrupts mitochondrial function and activates apoptosis in the hippocampus [10]. In spite of these findings, the effect of KD on brain function is controversial. While one study showed that rat fed with KD containing 42% of fat by energy with reduced carbohydrate impaired hippocampal neurogenesis [11], other study showed that such diet did not inhibit it [12]. Other study also showed that KD has a protective effect on kainic acid-induced cell death in the mouse hippocampus [13]. However, whether such diet affects the turnover of hippocampal neuron has not yet been clarified.

In the present study, we aimed to examine the effect of KD on cellular turnover in the dentate gyrus of the rat hippocampus. We also examined the combined effect with time-restricted diet (TRD), which is known to have beneficial effect in losing weight in humans [14] and rats [15], partly by reducing the amount of food intake [16]. TRD also prevents oxidative stress and memory impairments in the rat hippocampus [17]. Thus, we hypothesized that combination of KD with TRD may cause more beneficial effects in losing weight, accelerating neuronal turnover, and, if any, suppressing adverse effect of KD.

Cell apoptosis can be assessed by various methods. Among such methods, Caspase-3 is necessary for DNA fragmentation and morphological changes related to apoptosis [18]. On the other hand, Ki-67 is an excellent marker for determining cellular proliferation. The Ki-67 protein presents in the cells during all active phases of the cell cycle (G1, S, G2, and mitosis), but is absent in resting cells (G0) [19]. Thus, we studied the effects of the KD on the neuronal turnover in the dentate gyrus of the male Wistar rats with immunohistochemistry staining by using anti-Ki-67 and anti-caspase-3 antibodies.

## Methods

### Animals

Male Wistar rats (3 weeks old) weighing 60–70 g were purchased from Animal Laboratory, Biomedical Program, Faculty of Medicine, Universitas Sriwijaya (*n* = 24). They were housed under controlled temperature (22 °C) and illumination (12 h light/dark cycle: light on 6:00). Food and water were available ad libitum until the onset of the experiment. When we started the experiment, rats were divided into four groups (six rats/group): control, ketogenic diet (KD), time-restricted diet (TRD), and combination of ketogenic diet and time-restricted diet (CD) groups.

Animal experiments were performed according to the guidelines for the design and statistical analysis of experiments using laboratory animals after being approved by The Health Research Ethics Committee of the Faculty of Medicine, Universitas Sriwijaya, protocol number: 289/kepk/fkunsri/2019.

### Dietary methods

After the habituation of animals for 14 days, we started the experiment. All animals had free access to water throughout the experiment. The control group consumed standard pellets derived from Rat Bio 22/BR II, produced by PT. Japfa Comfeed Sidoarjo^®^, Indonesia. It consisted of 56% carbohydrate, 23.5% protein, 6.5% of fat, and 14% of others (1620 kcal/kg). KD group consumed high-fat nutrition, which consisted of 4.9% carbohydrate, 48.7% protein, and 46.35% fat (2195 kcal/kg). KD, which was processed in our institution, was made of egg yolks and chicken liver. TRD had freely access to the standard pellets for 8 h per day (12:00 to 20:00). Fasting time was determined according to a previous study [16]. CD group consumed KD for 8 h per day (12:00 to 20:00). The intervention was continued for 3 weeks. Daily food consumption was monitored and food intake information was analyzed with Nutrisurvey software to measure the intake of energy and nutrients. Diet methods are presented in Table 1.

**Table 1:** Diet methods

Group	Control	KD	TRD	CD
Feeding method	Ad libitum	Ad libitum	Ad libitum	Ad libitum
Feeding time	No restriction	No restriction	Restricted to 8 h (20:00–12:00)	Restricted to 8 h (20:00–12:00)
Provided food	Regular pellet	Ketogenic diet	Regular pellet	Ketogenic diet
Food consumption (g/rat/day)	25 ± 8	27 ± 10	19 ± 8	19 ± 9
Food consumption (kcal/rat/day)	40.5 ± 13	59.6 ± 20.1 l*	30.8 ± 13*	41.7 ± 19.7

Shown are mean ± SD. One-way ANOVA test for diet volume: [*F*(3,20) = 4.195; *P* < 0.05], *Bonferroni post hoc test: significant difference only between TRD vs. KD groups

### Ketone analysis

Before intervention and termination of the intervention (3 weeks after the onset of intervention), blood samples to measure ketone levels were collected from lateral tail vein. Rats were placed in a comfortable and unstressed position, and the blood was taken by making a small incision to the lateral tail vein using a razor blade. A drop of blood was placed on the Abbot Freestyle Optium Neo^®^ ketone meter (Abbot Indonesia, Jakarta, Indonesia).

### Brain tissue sampling

After the blood sampling, rats were sacrificed by cervical dislocation in the afternoon (between 15:00 and 17:00). Brain was taken, then fixed in 10% formalin, and embedded in paraffin. The block was then sectioned (3–4 μm thick) with a microtome, mounted on a glass slides, deparaffinized, and used for histological analysis.

### Immunohistochemistry

After deparaffinization, endogenous peroxidase was blocked by immersing in 0.5% H_2_O_2_ solution in methanol for 30 min, and then washed with running water for 5 min. Slides were immersed in Target Retrieval Solution (TRS) and heated in a microwave (100 W) until it boils, followed by a second heating using a lower power (10 W power level 1) for 5 min. Then they were left at room temperature until it cools down (about 5 min). Then the slides were washed with phosphate-buffered saline (PBS, pH 7.2–7.4) 3 times, each for 5 min.

The sections were circled with a PAP pen, dropped with a background sniper (ScyTek, Logan, Utah, USA), and incubated for 15 min. The tissue was incubated for one hour with rabbit anti-Ki-67 antibody (ready to use; ScyTek) and the other slide with rabbit anti-Caspase-3 antibody (ready to use, Bioss, Woburn, MA, USA) in the humidity chamber at room temperature; afterward, the slides were washed in PBS solution pH 7.2–7.4 3 times, each for 5 min. Slide was dripped with Ultra Tek HRP link, left for 20 min, and then washed in PBS solution pH 7.2–7.4 3 times, each for 5 min. Next, the slide was dripped with the Avidin Trek solution (HRP enzyme)-conjugated streptavidin and left for 20 min and then washed in PBS solution pH 7.2–7.4 3 times, each for 5 min. The tissue was then dripped with 1 ml substrate Betazid DAB buffer with 1 drop DAB Betazid chromogen (BiocareMedical, USA), and left for 2–10 min while being seen under a microscope until it turns brown and then rinsing in running water. Then the preparation was dipped in a solution of Mayer’s Hematoxylin for 1–2 min for counterstaining, and then washed in running water for 10 min. The slides were dipped with 5% saturated lithium carbonate (LiCO_3_) solution immersed in aquadest 2–3 times and washed in running water. Then sections were dehydrated in multilevel alcohol (96% alcohol for 5 min, then 100% alcohol 2 times, each for 5 min) followed by a clearing process in xylol 2 times, each for 5 min, dropped with mounting medium, and coverslipped.

### Quantitative analysis

ImageJ software was used to calculate the expression of Ki-67 and Caspase-3 in the dentate gyrus of the hippocampus. Each cell was traced and counted, started from dorsal to ventral. To avoid counting non-specific staining, the positive staining of the two antibodies was counted in the cells with intermediate and strong intensity. The label of each slide was masked so that examiner cannot discriminate the groups of each slide. All region was identified with 10 × magnification. Then, regions of interest (ROI) were captured at higher magnification (40 ×) to calculate the total number of nuclei/cells and the number of positive nuclei expressing Ki-67 or Caspase-3. The results were counted by percentage of positive nuclei/cells divided by total number of nuclei/cells. The final counts were determined from average calculation of 3 examiners.

### Statistical analysis

A one-way ANOVA test was used to analyze differences in Ki-67 and Caspase-3 levels of the four groups of rats, and an ANCOVA test was used to analyze body weight and blood ketone levels, as all of the numeric data exhibited normality and homogeneity in the variance with *P* > 0.05. For post hoc analyses, a Bonferroni test was used to assess the degree of difference among each group. The data analysis was performed in SPSS version 13.0 for Windows.

## Results

### Animal body weight

**Fig. 1:**
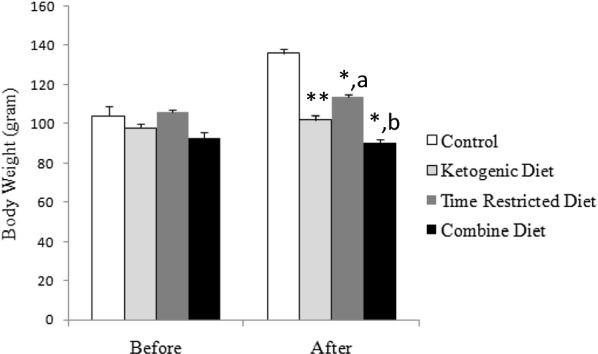
The effects of diet on body weight. Shown are mean ± SD. An ANCOVA test shows statistically significant [*F*(3,20) = 5.64; *P* = 0.006]. Significant differences among groups derived from Bonferroni post hoc tests are shown. ** and *: *P* < 0.01 and *P* < 0.05 vs. control, respectively. a: *P* < 0.05 vs. ketogenic diet. b: *P* < 0.05 vs. time-restricted diet. Note that no statistical difference was observed between ketogenic diet and combine diet groups

The effect of intervention of diet on body weight is illustrated in Fig. 1. Approximately 30% weight gain was observed 3 weeks after the intervention in the control groups. Such increase was not observed in the KD group (*P* < 0.01, vs. Control, by Bonferroni test), although the amount of food consumption is not different from those of the control group (Table 1). The weight gain was also significantly lower in the TRD group (*P* < 0.05, vs. Control, by Bonferroni test). No weight gain was observed by the combination of KD and TRD in the CD group (*P* < 0.05, vs. Control, by Bonferroni test). These results indicate that both KD and TRD are effective in inhibiting body weight gain.

### Blood ketone levels

**Fig. 2:**
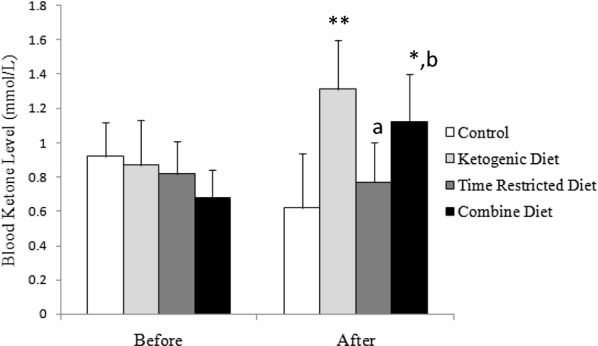
The effects of diet on blood ketone level. Shown are mean ± SD. An ANCOVA test shows statistically significant [*F*(3,20) = 5.64; *P* = 0.006]. Significant differences among groups derived from Bonferroni post hoc tests are shown. ** and *: *P* < 0.01 and *P* < 0.05 vs. control, respectively. a: *P* < 0.05 vs. ketogenic diet. b: *P* < 0.05 vs. time-restricted diet. Note that no statistical difference was observed between ketogenic diet and combine diet groups

The effects of intervention of diet on changes in blood ketone levels are illustrated in Fig. 2. The KD group showed an increase in the blood ketone levels compared to the control group (*P* < 0.01), whereas such increase was not observed in the TRD group. However, TRD did not normalize the increase in ketone level induced by KD as shown in the CD group (*P* < 0.05 vs. Control).

### The change in neurogenesis in the dentate gyrus

**Fig. 3:**
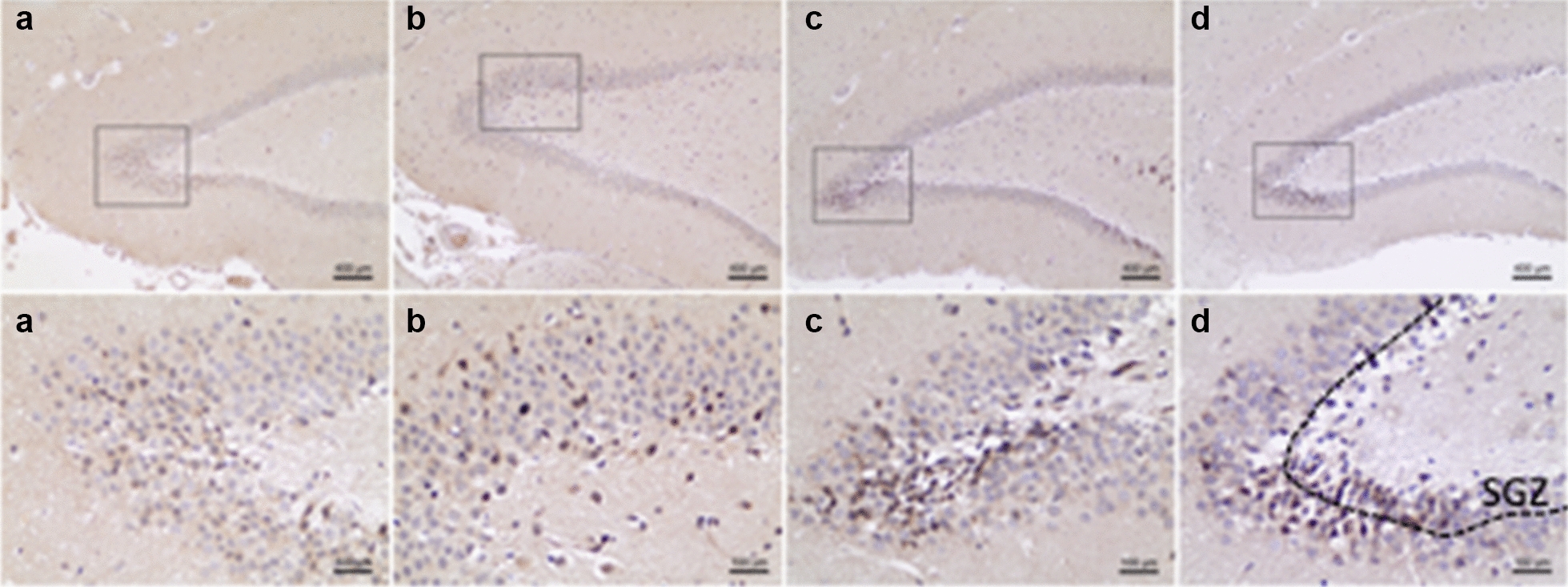
Immunohistochemistry of Ki-67 in the dentate gyrus. Upper panels show the localization of the region shown in the lower panel. Nuclei with KI-67-positive were mainly detected in the subgranular zone (SGZ) of the dentate gyrus, which is shown in the lower panel of **d**. Brown nuclear stain highlights KI-7-positive cells. The Ki-67-positive nuclei were seen more in the TRD (**c**) and CD (**d**) groups, and the cells were less seen in the control (a) and KD (b) groups

**Fig. 4:**
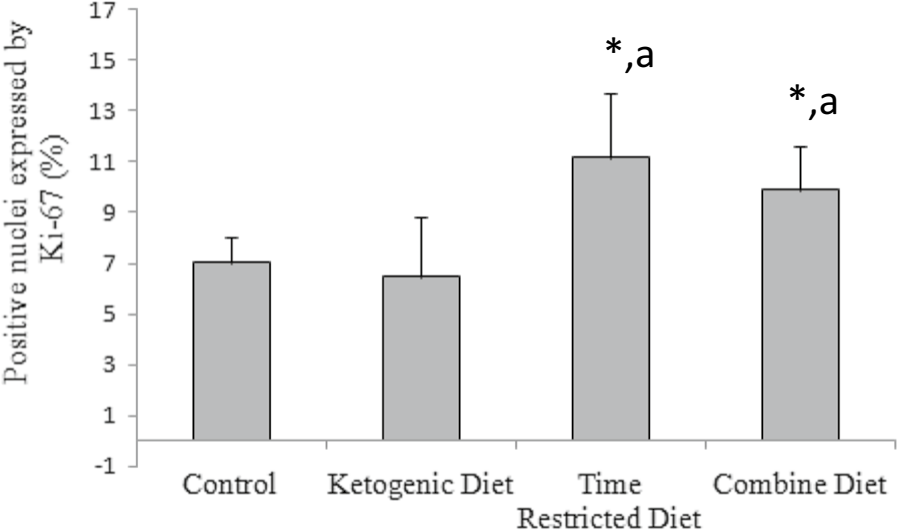
Percentage of positive nuclei expressing Ki-67 in the dentate gyrus of the hippocampus. A one-way ANOVA test shows a significant treatment effect in Ki 67 expression levels [*F*(3,20) = 11.086; *P* < 0,05]. Significant differences among groups derived from Bonferroni post hoc tests are shown. *: *P* < 0.05 vs. Control. a: *P* < 0.05 vs. ketogenic diet. There was no significant differences between control and ketogenic diet groups, and time-restricted diet and combine diet groups

Ki-67 level was used to measure the neurogenesis in the dentate gyrus of the hippocampus (Fig. 3). Ki-67 immunoreactive cells were distributed in the dentate gyrus with different densities among groups. The change in Ki-67’s expression was assessed by calculating the percentage of positive nuclei in dentate gyrus. While no difference in the expression levels of Ki-67 was observed between the control and KD group (*P* > 0.05), TRD showed an increase in the expression (*P* < 0.05 vs. Control) (Fig. 4). Such increase was also seen in the CD group (*P* < 0.05 vs. Control), indicating that KD did not affect TRD-activated neurogenesis.

### Alteration of apoptosis in the dentate gyrus

**Fig. 5:**
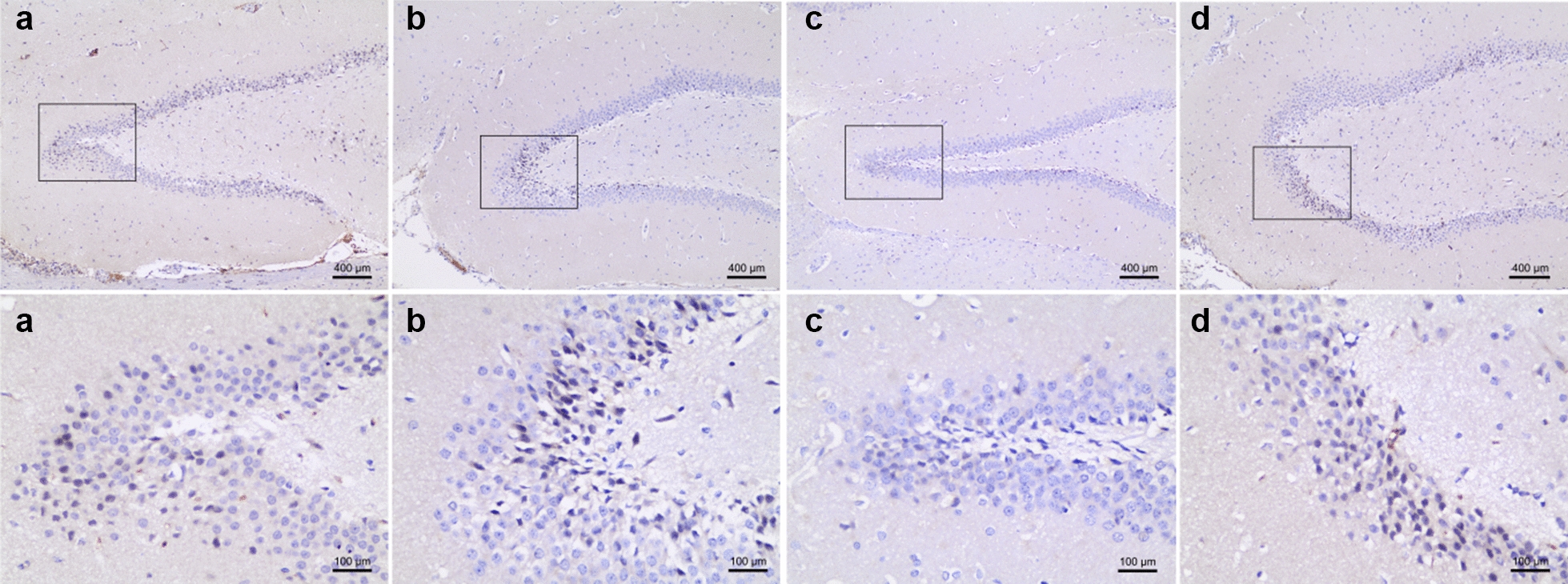
Immunohistochemistry of Caspase-3 in the dentate gyrus. Upper panels show the localization of the regions shown in the lower panel. The Caspase-3-positive cells were less seen in TRD (**c**) group compared to control (**a**) group. The Caspase-3-positive cells were more seen in KD (**c**) and CD (**d**) groups

**Fig. 6:**
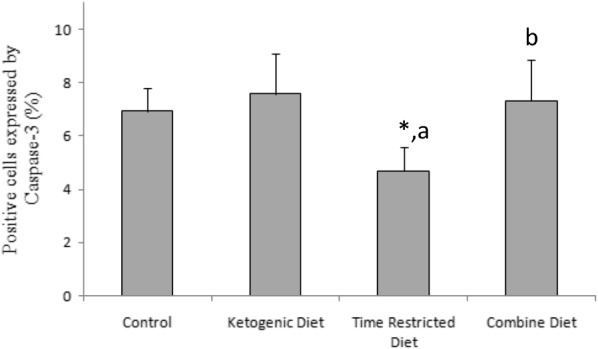
Percentage of positive cells expressing Caspase-3 in the dentate gyrus of the hippocampus. A one-way ANOVA test shows a significant treatment effect in Caspase-3 expression [*F*(3,20) = 6.616; *P* < 0.05]. Significant differences between groups derived from Bonferroni post hoc are shown. *: *P* < 0.05 vs. control. a: *P* < 0.05 vs. ketogenic diet. b: *P* < 0.05 vs. time-restricted diet. The percentage of positive cells was significantly lower in the TRD group than those in other groups

Apoptotic activity was detected by Caspase-3 staining (Fig. 5). Caspase-3-positive cells were mainly located in the subgranular zone of the dentate gyrus. As shown in Fig. 6, the KD did not alter the number of Capase-3-positive cells in this region (*P* > 0.05). However, TRD significantly decreases the number of Capase-3-positive cells (*P* < 0.05). No significant difference was observed between the CD and control groups, although CD group showed a higher number of Capase-3-positive cells compared with those of TRD group (*P* < 0.05). These results indicate that the decrease in apoptosis induced by TRD was cancelled by KD.

## Discussion

In this study, we have compared for the first time the effect of KD, TRD, and their combination altogether on neurogenesis and apoptosis in the dentate gyrus of the hippocampus. We have shown that the neurogenesis in the dentate gyrus measured by the expression of Ki-67 increased in the TRD and CD groups, whereas such difference was not observed in the KD group. The number of Capase-3-positive cells decreased significantly in the TRD group, whereas such decrease was not observed in the KD and CD groups, indicating that apoptosis was inhibited by TRD, but not by KD or CD. These results indicate that, although KD did not alter the neurogenesis and apoptosis in the dentate gyrus, it did not decrease the apoptosis seen in the TRD group. Furthermore, combination of TRD and KD eliminated the effect of TRD to decrease apoptotic cells. In addition, KD induced an increase in blood ketone level, which was not decreased by the combination of KD and TRD. Taken together, although KD could be effective in reducing the body weight, possible adverse effect in the brain cannot be fully ignored.

We have shown that KD, TRD, and CD groups were able to hold the weight compared to the control group. This result is consistent with a previous study in 8-week-old male mice showing a significant decrease in body weight after 5 weeks of treatment with a high fat and calorie restricted [20]. In our study, on the other hand, we started the experiment when rats were 5 weeks old, which were still in the stage of the growth and development. Thus, dietary intervention may have affected more greatly in their increase in body weight.

The diet implemented in the KD program mainly is a high-fat food with reduced amount of carbohydrates. The breakdown of fat deposits in adipose tissue due to an imbalance of calories in the body, causing lipolysis, and weight loss (holding the rate of body weight compared to control) [21]. KD with this diet method, the body’s energy source comes only from fat in the form of ketone bodies produced by liver cells [20]. A high-fat, low-carbohydrate diet can help control appetite and can increase the oxidative metabolism of fat which can cause weight loss [22]. KD used in the present study was also effective in suppressing the increase in body weight of the male rat, even though the calorie/kg pellet is higher than that of regular pellet. Furthermore, it should be noted that KD in the present study was more effective than TRD in reducing weight. In TRD group, food restriction was performed from 20:00 to 12:00 to mimic medical examinations of humans [16]. This time schedule may also mimic the feeding pattern during Ramadan of Muslims who do not eat during daytime. Taken together, our experiment could mimic the dietary intervention for humans, particularly for Muslims, and thus can be a useful model study to examine the effect of KD and/or combination of KD and TRD on various organ functions, including the brain.

In the present study, we found that blood ketone levels in the KD and CD were higher than those in the control group, although the ketone levels of the KD group did not exceed those in a previous study [23]. It is well known that the KD increases blood ketone levels compared to the normal diet, although the level does not reach to the metabolic ketoacidosis level [22]. The increase in blood ketones occurs as a result of shifting energy source from carbohydrates to fat, increasing the synthesis of ketone bodies as an alternative energy [24]. At present, there are no data regarding the maximum tolerance levels of ketones in rat. In humans, blood ketone levels in a healthy state during physiological ketosis do not exceed 8 mmol/L, because the brain can efficiently utilize ketone bodies to replace glucose [22]. However, although the ketone level is not as high as those during ketoacidosis, whether relatively high levels of ketones affect the brain function is not known. In the present study, we did not observe any difference in neurogenesis and apoptosis in the dentate gyrus between the control and KD groups, indicating that increased ketone level may have little effect on such processes. However, because we did not examine the cognitive function of our animal model, possibility of impaired neuronal plasticity in the hippocampus cannot be ignored. Further study may be required to study the effect of KD on the hippocampal function.

TRD-induced decrease in apoptosis was eliminated by KD. TRD is also well-recognized dietary intervention [3] and known to prevent oxidative stress and memory impairments in the rat hippocampus [17]. Although the effect of TRD on apoptosis in the dentate gyrus has not been reported, caloric restriction can decrease apoptosis by inhibiting of TRPV1 channel [25]. Thus, TRD-induced caloric restriction seen in the present study may have induced a decrease in apoptosis. TRD also induces transcription factors to express brain-derived neurotrophic factor (BDNF) [23], which plays a critical role on neuronal plasticity [26]. TRD also increases β-hydroxybutyrate (BHB) in the astrocyte [27]. BHB was re-utilized in mitochondrial biogenesis and synaptic plasticity [23]. Thus, the elimination of the decrease in apoptosis by TRD by KD may indicate that the increase in ketone levels may affect the brain function by altering such processes. Furthermore, KD may affect not only TRD-induced alteration of brain function but also other stimulations from the environment. Further study may be also required to examine the effect of KD on various stimulations from the environment.

## Conclusion

Ketogenic diet was effective to suppress the increase in the body weight in male rat. However, it elevated blood ketone levels. Although this intervention did not affect the neurogenesis in the dentate gyrus of the hippocampus, it suppressed the decreased apoptosis induced by time-restricted diet, which is known to be beneficial to brain function. Although the evident adverse effect of ketogenic diet on brain function has not yet been reported, the extreme high-fat with low-calorie diet may better to be avoided.

## Data Availability

The corresponding author has data that support the findings of this study. Data available upon reasonable request.

## Abbreviations

BMI: Body mass index
DNA: Deoxyribonucleic acid
H2O2: Hydrogen peroxide
TRS: Target retrieval solution
PBS: Phosphate-buffered saline
LiCO_3_: Lithium carbonate
BDNF: Brain-derived neurotrophic factor
